# Supplementary material for: The causal role of circulating inflammatory markers in osteoporosis: a bidirectional Mendelian randomized study
Source: Front Immunol. 2024 Jul 18;15:1412298. doi: 10.3389/fimmu.2024.1412298 (PMC11291241; doi:10.3389/fimmu.2024.1412298)
Supplement: Supplementary file 1 [file DataSheet_1.zip › supplementary material/CIM-OSTEOPOROSIS /GCST90274817.csv_leave_one_out.pdf]

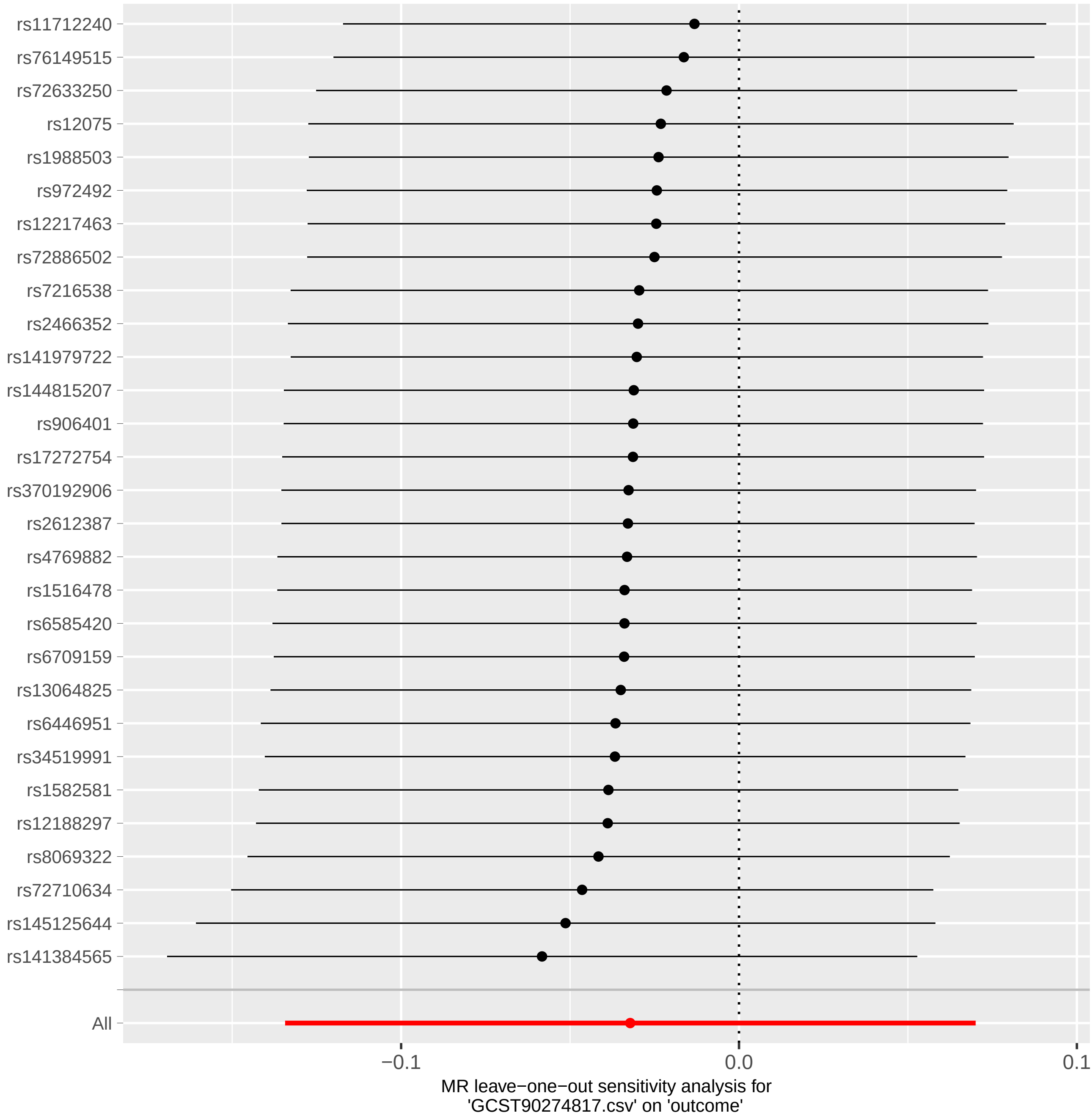
rs11712240
rs76149515
rs72633250
rs12075
rs1988503
rs972492
rs12217463
rs72886502
rs7216538
rs2466352
rs141979722
rs144815207
rs906401
rs17272754
rs370192906
rs2612387
rs4769882
rs1516478
rs6585420
rs6709159
rs13064825
rs6446951
rs34519991
rs1582581
rs12188297
rs8069322
rs72710634
rs145125644
rs141384565
All
−0.1
0.0
0.1
MR leave−one−out sensitivity analysis for
'GCST90274817.csv' on 'outcome'
